# Notes and News

**Published:** 1886-10-30

**Authors:** 


### Notes and News.

Collected and Communicated.

The article on the registration-fee system for out-patients, by Lord Leigh, which appears in another part of this issue, will direct attention to a subject the merits of which have often been discussed, but not, so far, definitely decided. The plan of taking a small registration-fee from out-patients has been introduced at Guy's Hospital, and also at the Children's and Women's Hospitals, Birmingham. It has possibly been tried elsewhere, and we shall be glad to receive particulars of the results at Guy's and wherever else it may have been introduced.

Cottage hospitals are springing up almost everywhere. Lord Brownlow having given a site, a new cottage hospital has just been erected at Whitchurch, Salop, from the plans of Mr. E. W. Mountford of Buckingham Street, Adelphi, W.C. The new hospital was opened a week ago, free from debt, and Lord Kenyon has liberally endowed it, so that Whitchurch will be well supplied with hospital accommodation in years to come.

A new Children's Hospital is about to be erected at Newcastle, through the munificence of Mr. Fleming. Is it too much to hope that some other wealthy residents will show an equal liberality, by contributing freely to the funds of the Newcastle Infirmary, which is at present suffering from great financial distress?

Dr. A. B. Low has been appointed physician to the Sunderland Infirmary, *vice* Dr. Dixon, resigned. Two features in connection with this election are noteworthy. The vacancy was filled up by an election committee chosen by the governors, so that the candidates were spared the expense and humiliation of a personal visit to all the governors, and canvassing of any kind was held to be a disqualification. What a change, and what an improvement upon the old system!

An honorary physician is required for the Children's Hospital at Newcastle, through the resignation of Dr. Houseman, and Dr. Heath's resignation has caused a vacancy in the office of surgeon to the Eye Infirmary. Four candidates are already in the field for the physicianship, namely, Drs. Beatley, Robertson, Baumgartner, and Blair. The contest is, we hear, likely to be keen and exciting, as the post is much coveted by the younger members of the profession in Newcastle.

The statue of Sister Dora at Walsall is of Sicilian marble, 7 feet 10 inches in height. It represents Sister Dora dressed as a hospital sister, in the act of unrolling a bandage. The statue has been placed on the bridge in the centre of the town, where it will be seen by all, whether strangers or residents.

It is remarkable to what a small extent a subscription-box in the out-patient department of a hospital or dispensary seems to appeal to the patients and their friends. A registration-fee will be cheerfully paid, and upwards of £800 a year may be realised from this source, as in the case of the Queen's Hospital, Birmingham, last year; but a subscription-box at the entrance to or in the hall of the out-patient departments of the largest hospitals in the country would probably produce less than a five-pound note per annum. At one of the dispensaries such a box was found to contain four shillings, although a charge of one penny for each medicine-bottle issued to the patients produced £40 a year. If the patients think this matter out for themselves, such subscription-boxes will doubtless contain more for the future.

The resident medical officer of the Miller Hospital and Royal Kent Dispensary having resigned, after many years' service, it has been determined to divide the duties and to appoint a qualified man to attend to the dispensary work, and a second to look after the hospital patients. This will no doubt be an improvement on the old system, as it will ensure that a doctor shall be at all times available within the hospital, in cases of accident or emergency.

The committee managing the affairs of the Miller Hospital at Greenwich have often shown great public spirit. At the outset they had the courage to enact that no one should be eligible for the post of surgeon to the hospital who was not a Fellow by examination of one of the Colleges, or a graduate in surgery, of a British University. More recently, under difficult circumstances, they have shown a discretion and courage worthy of high commendation. These remarks are called for by some anonymous correspondence which has been inserted in the *Greenwich Observer*, and which we know to be as ill-timed as the charges made are unfounded.

Major Tulloch, an inspector of the Local Government Board, has recommended the erection of a smallpox-hospital for the district of the Orsett Sanitary Authority, at an expense of £1,000. The new buildings are likely to be commenced next March.

There is a vacancy for an Honorary Assistant Physician at the City of London Hospital for Diseases of the Chest, Victoria Park, E. Applications must be made to the Secretary before the 11th Nov. next.

---

The following vacancies are announced this week, the amount of the salary being placed in each case after the name of the institution :—

**Resident Medical Officers.**

Ramsgate Infirmary, doubly qualified.—£130 without board.
Nottingham Dispensary, doubly qualified.—£150 per annum without board.
Liverpool Dispensary, 34, Moorfields, assistant, registered.—£80 without board.
Gravesend Hospital, surgical qualification.—£80 with board.
Rotherham Hospital, doubly qualified.—£100 with board.
Manchester Dispensary, 113, Market Street, doubly qualified.—£100 without board.
Bury Dispensary, Junior Medical Officer, doubly qualified.—£60 with board.
Hants County Asylum, junior assistant, doubly qualified.—£100 with board.

**Dispenser.**

Seamen's Hospital, Greenwich.—£70 with board.

**Matron and Housekeeper.**

York Lunatic Asylum.—£60.

**Trained Nurses.**

Braintree and Bocking Cottage Hospital, sole charge.—£27 12s.
Denbighshire Infirmary, Denbigh, head nurse, speaking Welsh.—£25. Also a probationer, £18.
Fever Hospital, Newcastle-on-Tyne, night nurse.—£25 to £35.

---

Women were re-admitted on the 21st instant, after an interval of twelve years, to the Extra-mural Medical School at Edinburgh. Six ladies who have recently passed the preliminary examinations in arts have entered as students for the classes. Dr. Sophia Jex Blake is the acting organising secretary, and the classes will be conducted by Dr. Macdonald Browne and Dr. Stevenson Macadam.

---

Sheffield has determined to have a new school of medicine, and £3,000 of the £6,000 required for the new buildings, was subscribed at the town's meeting, held at Firth College on the 22nd instant. The hospitals of Sheffield contain 300 beds, and afford an excellent field for the acquisition of medical and surgical knowledge. The proposal was supported by the Mayor of Sheffield, Alderman T. W. Pye Smith, the Archbishop of York, the Master Cutler, Mr. G. F. Lockwood, Sir Andrew Clark, M.D., Sir F. T. Mappin, M.P., and others. The proceedings were enthusiastic throughout, and the new Medical College promises to be a great success.

---

The British Medical Association will hold its annual meeting at Dublin in 1887, and Dr. J. T. Banks, Professor of Physiology at the University of Dublin, has been appointed president elect.

The eighth cremation at the Crematory of the Cremation Society of England, Woking, took place on the 21st inst. The remains were those of a lady, 64 years of age, whose wish it was to be thus disposed of after death. The process occupied more than an hour, and the ashes, of a pure white colour, weighing $3\frac{1}{4}$ lb., were taken possession of by the two sons, who attended the ceremony.

---

Church parades seem to be highly popular with the members of Friendly Societies. For some months past it is estimated that something like £100 a week has been raised for various hospitals through the agency of trades processions and special services.

---

Some irritation seems to have been caused at Stratford by the omission from the speeches, at the close of the bazaar, of all allusion to what the working men had done, by raising money for the proposed hospital. It may be as well, therefore, to repeat, that the West Ham Hospital was originated by the Friendly Societies, and that unless they had raised £500 and stuck manfully to the work, there would probably have been neither a bazaar nor a hospital, unless we are greatly misinformed.

---

On Saturday last His Grace the Roman Catholic Archbishop of Dublin, the Most Rev. Dr. Walsh, dedicated a new chapel, which has recently been erected from the plans of Mr. John L. Robinson, A.R.H.A., in connection with the Mater Misericordiæ Hospital, Dublin. This hospital was designed by the late Mr. John Burke, in the form of a hollow square. The original plan was not carried out at the time owing to a want of funds, and this first intention was afterwards wisely abandoned on hygienic grounds, when three sides of the square had been built. The new western wing, in which the chapel is situated, contains accommodation for fifty additional patients, as well as the chapel, and also a convent for the residence of sisters in charge of the hospital. The fifty beds are placed in eight wards, and are intended for the treatment of special cases. New bath-rooms with every convenience have likewise been added, and the whole of the handsome furniture for the chapel has been given anonymously by a benevolent lady.

---

The Bishop of Durham has doubled his subscription to the Newcastle Infirmary, which is much in need of increased support, providing the committee decide to maintain the work of the institution on its present scale. His Lordship adds, that he believes many others will thankfully do the same, and we are inclined to agree with him in his opinion. It is certain that the mere reduction of beds will cause an amount of unnecessary distress to the poor inhabitants of Newcastle, without reducing the expenditure to an amount in any way adequate to the misery such a step would cause. The closing of a few beds in the case of a large hospital cannot materially reduce expenditure, and in such cases the right course undoubtedly is to determine to bring to bear the necessary energy to secure such an increase of support as will make the income equal to defraying the cost of the claims made upon the managers by the suffering poor.

---

The professorship of Materia Medica at the Aberdeen University has been rendered vacant by the untimely death of Professor Dyce-Davidson. The late Professor was in his class-room in Marischal College last Friday afternoon, when a blood-vessel in the head suddenly burst. Death ensued in a very short time. Dr. Dyce-Davidson, who was only 41 years of age, was a native of Aberdeen. He graduated M.A. at the University in 1863, and took his M.D. degree in 1866. He was appointed Professor of Materia Medica in 1878.

---

Mr. Emil Behnke, in a little pamphlet entitled *Physician and Voice Trainer*, has replied dispassionately and ably to Dr. Morell Mackenzie's criticisms in his recent work on *Hygiene of the Vocal Organs*. Those who have read the work of this distinguished throat-surgeon, should not fail to read the very temperate answer which Mr. Behnke has written.

---

An inquest held on the 21st inst., as to the death of Emma Peek, the wife of a carpenter, who had been a patient in the Highgate Infirmary from the 10th August to the 11th of September, and who left because she complained to her husband of what she considered the cruel treatment she had received there, calls attention once more to the management of that institution. Within recent years we believe that there have been two Local Government Board inquiries, caused by the evidence given at two previous inquests upon patients who had died at the Highgate Infirmary. In Mrs. Peek's case, it is true, that no evidence was produced in support of the allegations as to any ill-treatment at Highgate, but the fact that she should have induced her husband to allow her to leave the infirmary for the reasons stated, coupled with the earlier circumstances to which we have alluded, point to the desirability of a thorough investigation into the administration of this institution by the committee of which Mr. W. Adams, F.R.C.S., is the able chairman.

Previous to the passing of Hardy's Act, twenty years ago, the management of the Poor-law Infirmaries throughout the metropolis was in a scandalous condition. Great improvements have taken place of late years, but these inquest cases are not reassuring. We purpose giving an account of the nursing and other arrangements at these institutions before long, so we shall not refer further to them to-day.

---

It would appear, from the report of Dr. Collie, the medical superintendent of the Eastern Hospital, that the number of cases of enteric fever under treatment, viz., 112, was last year less than in any year since the opening of the hospital in 1871. It would be interesting to know if there has been a similar decrease in these cases at all the fever hospitals throughout the metropolis during the year.

---

It has been decided to provide temporary hospital accommodation for infectious cases on land belonging to the Liverpool Corporation, adjoining the Netherfield Road Hospital, at a cost not exceeding £600. Dr. Palmer was appointed resident medical officer of this hospital at a meeting held on the 19th inst.

---

Miss Steele has been presented with a purse, together with a gold watch and chain, on resigning the matronship of the Isle of Man Hospital, in grateful recognition of her long services as matron, and of her kindness to the patients. We understand that the sickness of a near and dear relative is the cause of Miss Steele's resignation, and hopes were expressed at the meeting at which the presentation was made, that Miss Steele might be able one day to resume her duties in the island. It is gratifying to be able to record so substantial a recognition of able and faithful service on the part of a hospital matron.

---

There is no doubt that the provincial hospitals look to those in the metropolis for guidance on many points, but the London hospitals might learn something also from the institutions in the provinces. One point worthy of consideration and investigation is, how the provincial hospitals manage to maintain an occupied bed at so much less a sum than the cost of an occupied bed in the metropolis. The provincial hospitals further acknowledge, once a month or oftener, in the newspapers in a paragraph form, for which they pay, we believe, a relatively small sum, the larger donations and more valuable gifts in kind which they receive. It cannot be doubted, if some such arrangements could be made with the metropolitan press, that the London hospitals would greatly benefit by the increased publicity which would thus be secured.

